# Supplementary material for: The effects of genital myiasis on the diversity of the vaginal microbiota in female Bactrian camels
Source: BMC Vet Res. 2022 Mar 5;18:87. doi: 10.1186/s12917-022-03189-5 (PMC8897907; doi:10.1186/s12917-022-03189-5)
Supplement: Supplementary file 5 — Additional file 5. [file 12917_2022_3189_MOESM5_ESM.zip › MPL201709200_16s_yy/Treat1/css/js_css/jsPDF-master.zip!/jsPDF-master/docs/plugins_split_text_to_size.js.html]

plugins/split\_text\_to\_size.js - Documentation


## Home

### Classes

- jsPDF

### Global

- addFont
- addHTML
- addMetadata
- addPage
- autoPrint
- CapJoinStyles
- circle
- ellipse
- getFontList
- lines
- lstext
- output
- rect
- roundedRect
- save
- setDisplayMode
- setDrawColor
- setFillColor
- setFont
- setFontSize
- setFontStyle
- setLineCap
- setLineJoin
- setLineWidth
- setPage
- setProperties
- setTextColor
- text
- triangle

# plugins/split\_text\_to\_size.js

```
/** @preserve
 * jsPDF split_text_to_size plugin - MIT license.
 * Copyright (c) 2012 Willow Systems Corporation, willow-systems.com
 *               2014 Diego Casorran, https://github.com/diegocr
 */
/**
 * Permission is hereby granted, free of charge, to any person obtaining
 * a copy of this software and associated documentation files (the
 * "Software"), to deal in the Software without restriction, including
 * without limitation the rights to use, copy, modify, merge, publish,
 * distribute, sublicense, and/or sell copies of the Software, and to
 * permit persons to whom the Software is furnished to do so, subject to
 * the following conditions:
 *
 * The above copyright notice and this permission notice shall be
 * included in all copies or substantial portions of the Software.
 *
 * THE SOFTWARE IS PROVIDED "AS IS", WITHOUT WARRANTY OF ANY KIND,
 * EXPRESS OR IMPLIED, INCLUDING BUT NOT LIMITED TO THE WARRANTIES OF
 * MERCHANTABILITY, FITNESS FOR A PARTICULAR PURPOSE AND
 * NONINFRINGEMENT. IN NO EVENT SHALL THE AUTHORS OR COPYRIGHT HOLDERS BE
 * LIABLE FOR ANY CLAIM, DAMAGES OR OTHER LIABILITY, WHETHER IN AN ACTION
 * OF CONTRACT, TORT OR OTHERWISE, ARISING FROM, OUT OF OR IN CONNECTION
 * WITH THE SOFTWARE OR THE USE OR OTHER DEALINGS IN THE SOFTWARE.
 * ====================================================================
 */

;(function(API) {
'use strict'

/**
Returns an array of length matching length of the 'word' string, with each
cell ocupied by the width of the char in that position.

@function
@param word {String}
@param widths {Object}
@param kerning {Object}
@returns {Array}
*/
var getCharWidthsArray = API.getCharWidthsArray = function(text, options){

	if (!options) {
		options = {}
	}

	var widths = options.widths ? options.widths : this.internal.getFont().metadata.Unicode.widths
	, widthsFractionOf = widths.fof ? widths.fof : 1
	, kerning = options.kerning ? options.kerning : this.internal.getFont().metadata.Unicode.kerning
	, kerningFractionOf = kerning.fof ? kerning.fof : 1

	// console.log("widths, kergnings", widths, kerning)

	var i, l
	, char_code
	, prior_char_code = 0 // for kerning
	, default_char_width = widths[0] || widthsFractionOf
	, output = []

	for (i = 0, l = text.length; i < l; i++) {
		char_code = text.charCodeAt(i)
		output.push(
			( widths[char_code] || default_char_width ) / widthsFractionOf +
			( kerning[char_code] && kerning[char_code][prior_char_code] || 0 ) / kerningFractionOf
		)
		prior_char_code = char_code
	}

	return output
}
var getArraySum = function(array){
	var i = array.length
	, output = 0
	while(i){
		;i--;
		output += array[i]
	}
	return output
}
/**
Returns a widths of string in a given font, if the font size is set as 1 point.

In other words, this is "proportional" value. For 1 unit of font size, the length
of the string will be that much.

Multiply by font size to get actual width in *points*
Then divide by 72 to get inches or divide by (72/25.6) to get 'mm' etc.

@public
@function
@param
@returns {Type}
*/
var getStringUnitWidth = API.getStringUnitWidth = function(text, options) {
	return getArraySum(getCharWidthsArray.call(this, text, options))
}

/**
returns array of lines
*/
var splitLongWord = function(word, widths_array, firstLineMaxLen, maxLen){
	var answer = []

	// 1st, chop off the piece that can fit on the hanging line.
	var i = 0
	, l = word.length
	, workingLen = 0
	while (i !== l && workingLen + widths_array[i] < firstLineMaxLen){
		workingLen += widths_array[i]
		;i++;
	}
	// this is first line.
	answer.push(word.slice(0, i))

	// 2nd. Split the rest into maxLen pieces.
	var startOfLine = i
	workingLen = 0
	while (i !== l){
		if (workingLen + widths_array[i] > maxLen) {
			answer.push(word.slice(startOfLine, i))
			workingLen = 0
			startOfLine = i
		}
		workingLen += widths_array[i]
		;i++;
	}
	if (startOfLine !== i) {
		answer.push(word.slice(startOfLine, i))
	}

	return answer
}

// Note, all sizing inputs for this function must be in "font measurement units"
// By default, for PDF, it's "point".
var splitParagraphIntoLines = function(text, maxlen, options){
	// at this time works only on Western scripts, ones with space char
	// separating the words. Feel free to expand.

	if (!options) {
		options = {}
	}

	var line = []
	, lines = [line]
	, line_length = options.textIndent || 0
	, separator_length = 0
	, current_word_length = 0
	, word
	, widths_array
	, words = text.split(' ')
	, spaceCharWidth = getCharWidthsArray(' ', options)[0]
	, i, l, tmp, lineIndent

	if(options.lineIndent === -1) {
		lineIndent = words[0].length +2;
	} else {
		lineIndent = options.lineIndent || 0;
	}
	if(lineIndent) {
		var pad = Array(lineIndent).join(" "), wrds = [];
		words.map(function(wrd) {
			wrd = wrd.split(/\s*\n/);
			if(wrd.length > 1) {
				wrds = wrds.concat(wrd.map(function(wrd, idx) {
					return (idx && wrd.length ? "\n":"") + wrd;
				}));
			} else {
				wrds.push(wrd[0]);
			}
		});
		words = wrds;
		lineIndent = getStringUnitWidth(pad, options);
	}

	for (i = 0, l = words.length; i < l; i++) {
		var force = 0;

		word = words[i]
		if(lineIndent && word[0] == "\n") {
			word = word.substr(1);
			force = 1;
		}
		widths_array = getCharWidthsArray(word, options)
		current_word_length = getArraySum(widths_array)

		if (line_length + separator_length + current_word_length > maxlen || force) {
			if (current_word_length > maxlen) {
				// this happens when you have space-less long URLs for example.
				// we just chop these to size. We do NOT insert hiphens
				tmp = splitLongWord(word, widths_array, maxlen - (line_length + separator_length), maxlen)
				// first line we add to existing line object
				line.push(tmp.shift()) // it's ok to have extra space indicator there
				// last line we make into new line object
				line = [tmp.pop()]
				// lines in the middle we apped to lines object as whole lines
				while(tmp.length){
					lines.push([tmp.shift()]) // single fragment occupies whole line
				}
				current_word_length = getArraySum( widths_array.slice(word.length - line[0].length) )
			} else {
				// just put it on a new line
				line = [word]
			}

			// now we attach new line to lines
			lines.push(line)
			line_length = current_word_length + lineIndent
			separator_length = spaceCharWidth

		} else {
			line.push(word)

			line_length += separator_length + current_word_length
			separator_length = spaceCharWidth
		}
	}

	if(lineIndent) {
		var postProcess = function(ln, idx) {
			return (idx ? pad : '') + ln.join(" ");
		};
	} else {
		var postProcess = function(ln) { return ln.join(" ")};
	}

	return lines.map(postProcess);
}

/**
Splits a given string into an array of strings. Uses 'size' value
(in measurement units declared as default for the jsPDF instance)
and the font's "widths" and "Kerning" tables, where available, to
determine display length of a given string for a given font.

We use character's 100% of unit size (height) as width when Width
table or other default width is not available.

@public
@function
@param text {String} Unencoded, regular JavaScript (Unicode, UTF-16 / UCS-2) string.
@param size {Number} Nominal number, measured in units default to this instance of jsPDF.
@param options {Object} Optional flags needed for chopper to do the right thing.
@returns {Array} with strings chopped to size.
*/
API.splitTextToSize = function(text, maxlen, options) {
	'use strict'

	if (!options) {
		options = {}
	}

	var fsize = options.fontSize || this.internal.getFontSize()
	, newOptions = (function(options){
		var widths = {0:1}
		, kerning = {}

		if (!options.widths || !options.kerning) {
			var f = this.internal.getFont(options.fontName, options.fontStyle)
			, encoding = 'Unicode'
			// NOT UTF8, NOT UTF16BE/LE, NOT UCS2BE/LE
			// Actual JavaScript-native String's 16bit char codes used.
			// no multi-byte logic here

			if (f.metadata[encoding]) {
				return {
					widths: f.metadata[encoding].widths || widths
					, kerning: f.metadata[encoding].kerning || kerning
				}
			}
		} else {
			return 	{
				widths: options.widths
				, kerning: options.kerning
			}
		}

		// then use default values
		return 	{
			widths: widths
			, kerning: kerning
		}
	}).call(this, options)

	// first we split on end-of-line chars
	var paragraphs
	if(Array.isArray(text)) {
		paragraphs = text;
	} else {
		paragraphs = text.split(/\r?\n/);
	}

	// now we convert size (max length of line) into "font size units"
	// at present time, the "font size unit" is always 'point'
	// 'proportional' means, "in proportion to font size"
	var fontUnit_maxLen = 1.0 * this.internal.scaleFactor * maxlen / fsize
	// at this time, fsize is always in "points" regardless of the default measurement unit of the doc.
	// this may change in the future?
	// until then, proportional_maxlen is likely to be in 'points'

	// If first line is to be indented (shorter or longer) than maxLen
	// we indicate that by using CSS-style "text-indent" option.
	// here it's in font units too (which is likely 'points')
	// it can be negative (which makes the first line longer than maxLen)
	newOptions.textIndent = options.textIndent ?
		options.textIndent * 1.0 * this.internal.scaleFactor / fsize :
		0
	newOptions.lineIndent = options.lineIndent;

	var i, l
	, output = []
	for (i = 0, l = paragraphs.length; i < l; i++) {
		output = output.concat(
			splitParagraphIntoLines(
				paragraphs[i]
				, fontUnit_maxLen
				, newOptions
			)
		)
	}

	return output
}

})(jsPDF.API);
```

  

Documentation generated by JSDoc 3.4.2 on Sun Oct 09 2016 11:08:27 GMT+0100 (BST) using the docdash theme.
